# Is there a sex-shift in prevalence of allergic rhinitis and comorbid asthma from childhood to adulthood? A meta-analysis

**DOI:** 10.1186/s13601-017-0176-5

**Published:** 2017-12-05

**Authors:** M. Fröhlich, M. Pinart, T. Keller, A. Reich, B. Cabieses, C. Hohmann, D. S. Postma, J. Bousquet, J. M. Antó, T. Keil, S. Roll

**Affiliations:** 1 Institute of Social Medicine, Epidemiology and Health Economics, Charité - Universitätsmedizin Berlin, Berlin, Germany; 2 Clinic for Neonatology, Charité - Universitätsmedizin Berlin, Berlin, Germany; 3 Max-Delbrück-Centrum für Molekulare Medizin, Research Team Molecular Epidemiology, Berlin, Germany; 4 ISGlobal, Centre for Research in Environmental Epidemiology (CREAL), Barcelona, Spain; 5 IMIM (Hospital del Mar Research Institute), Barcelona, Spain; 6 Universitat Popmpeu Fabra (UPF), Barcelona, Spain; 7 CIBER Epidemiología y Salud Pública (CIBERESP), Barcelona, Spain; 8 Epidemiology, German Rheumatism Research Centre, Berlin, Germany; 9 Facultad de Medicina Clínica Alemana, Universidad del Desarrollo, Santiago, Chile; 10 Department of Pulmonology, University Medical Center Groningen, University of Groningen, Groningen, The Netherlands; 11 University Hospital, Montpellier, France; 12 MACVIA-LR, Contre les Maladies Chroniques pour un Vieillissement Actifen Languedoc Roussillon, European Innovation Partnership on Active and Healthy Ageing Reference Site, and INSERM, VIMA: Ageing and Chronic Diseases, Epidemiological and Public Health Approaches, U1168, Paris, France; 13 UVSQ, UMR-S 1168, Université Versailles, St-Quentin-en-Yvelines, France; 14 Institute of Clinical Epidemiology and Biometry, University of Wuerzburg, Würzburg, Germany

**Keywords:** Allergic rhinitis, Asthma, Multimorbidity, Prevalence, Systematic review

## Abstract

**Background:**

Allergic rhinitis and asthma as single entities affect more boys than girls in childhood but more females in adulthood. However, it is unclear if this prevalence sex-shift also occurs in allergic rhinitis and concurrent asthma. Thus, our aim was to compare sex-specific differences in the prevalence of coexisting allergic rhinitis and asthma in childhood, adolescence and adulthood.

**Methods:**

Post-hoc analysis of systematic review with meta-analysis concerning sex-specific prevalence of allergic rhinitis. Using random-effects meta-analysis, we assessed male–female ratios for coexisting allergic rhinitis and asthma in children (0–10 years), adolescents (11–17) and adults (> 17). Electronic searches were performed using MEDLINE and EMBASE for the time period 2000–2014. We included population-based observational studies, reporting coexisting allergic rhinitis and asthma as outcome stratified by sex. We excluded non-original or non-population-based studies, studies with only male or female participants or selective patient collectives.

**Results:**

From a total of 6539 citations, 10 studies with a total of 93,483 participants met the inclusion criteria. The male–female ratios (95% CI) for coexisting allergic rhinitis and asthma were 1.65 (1.52; 1.78) in children (N = 6 studies), 0.61 (0.51; 0.72) in adolescents (N = 2) and 1.03 (0.79; 1.35) in adults (N = 2). Male–female ratios for allergic rhinitis only were 1.25 (1.19; 1.32, N = 5) in children, 0.80 (0.71; 0.89, N = 2) in adolescents and 0.98 (0.74; 1.30, N = 2) in adults, respectively.

**Conclusions:**

The prevalence of coexisting allergic rhinitis and asthma shows a clear male predominance in childhood and seems to switch to a female predominance in adolescents. This switch was less pronounced for allergic rhinitis only.

**Electronic supplementary material:**

The online version of this article (10.1186/s13601-017-0176-5) contains supplementary material, which is available to authorized users.

## Background

Increasing prevalence in allergic diseases has been observed in many countries, especially in Western but also many developing countries [1]. Sex specific differences in prevalence of allergic rhinitis and asthma over the life span were recognized, showing a higher prevalence of allergic rhinitis and asthma as single entities in boys than in girls during childhood followed by an equal distribution in adolescence [2, 3]. In adulthood more women than men are affected by asthma [4, 5]. In a prospective cohort study, the prevalence of coexisting eczema, allergic rhinitis, and asthma in the same child was more common than expected by chance alone and was not only attributable to IgE sensitization, suggesting that these diseases share causal mechanisms [6]. In a systematic review of studies across the globe we showed a sex-switch in prevalence of allergic rhinitis in population-based studies [3]. Since research on multimorbidity, i.e. the coexistence of 2 or more allergic diseases in the same individual, is sparse, the aim of this systematic review with meta-analyses was to examine sex specific differences in the prevalence of coexisting allergic rhinitis and asthma, from childhood through adolescence into adulthood.

## Methods

### Data sources, search strategy, and selection criteria

We conducted a systematic literature search using the online databases MEDLINE and EMBASE. MeSH terms were used in conjunction with keywords searched in the title and abstract. We restricted our search to studies published between January 2000 and April 2014. There was no restriction to the language of publication. The protocol for our systematic review was developed with guidance from the Preferred Reporting Items for Systematic Review and Meta-Analysis (PRISMA) statement [7]. It can be accessed at PROSPERO (http://www.crd.york.ac.uk/PROSPERO/, registration number CRD42016036105). To manage the identified publications, we used EndNote X7^®^ (Thomson Reuters) bibliographic database.

### Inclusion and exclusion criteria

The selection of studies was performed along with pre-set criteria for in- or exclusion. Since the present study is a post hoc analysis of a larger review considering the difference in prevalence for allergic rhinitis only [3], we chose broad inclusion criteria to reach most of the available information and to increase generalisability. The present analysis included studies of the previous comprehensive review that (1) recruited participants of both sexes from the general population, (2) reported the prevalence of coexisting allergic rhinitis and asthma, asthma only, and allergic rhinitis only stratified by sex and age if the population under study included both children and adults, and (3) were designed as longitudinal or cross-sectional studies.

We excluded (1) non-original studies (e.g. reviews or guidelines), (2) studies that selected participants by special occupation, (3) studies with only male or female participants, (4) studies analysing selective patient collectives (e.g. from special allergy clinics), or (5) non-population-based study designs e.g. ecological studies, case reports, case series, case–control studies, experimental studies, intervention studies, and clinical studies.

We evaluated prevalence estimates of allergic rhinitis, asthma and coexisting allergic rhinitis and asthma regarding the following endpoints: allergic rhinitis only was defined as having symptoms of allergic rhinitis (i.e. runny nose without having a cold) without having symptoms of asthma. In analogy, asthma only was defined as having symptoms of asthma (i.e. wheezing or whistling in the chest) but no symptoms of allergic rhinitis. An individual who named both symptoms of allergic rhinitis and symptoms of asthma was included in the group of coexisting allergic rhinitis and asthma. If selected studies reported prevalence rates for having symptoms ever or current, we chose ‘current’, which was defined as reporting symptoms in the last 12 months.

### Study selection, data extraction and quality assessment

A detailed protocol of the selection process for the initial review was published elsewhere [3]. In short a two-step review process was performed with scanning titles of identified studies first independently by two reviewers (MP and CH), followed by a second screening of all abstracts of articles rated as ‘include’ or ‘unclear’. A disagreement between the two reviewers was resolved by discussion to meet a consensus. If consensus was not reached, a third independent reviewer (TKei or MF) was asked to assess the relevance.

Prior to data extraction, two reviewers (MP and MF) independently reviewed full texts of all selected publications rated as ‘include’ or ‘unclear’. A pre-designed data extraction form was piloted with five studies selected from the pool of included studies. At least two reviewers (TKel, CH, BC, TKei, AR, MP and MF) extracted data from the selected full texts independently with disagreements through referral to a third reviewer (TKei).

For data extraction we used a self-designed (MP) SoSci-Survey questionnaire (https://www.soscisurvey.de/) retrieving information on country, study design, description of the process of recruitment of participants, age of participants, sample size, residency, response rate, observation period, definition of disease and measurement, method of data collection, prevalence of allergic rhinitis only (i.e. subjects without asthma) and asthma only (i.e. subjects without allergic rhinitis) as well as coexisting allergic rhinitis and asthma stratified by sex. Prevalences for each study were calculated using the number of participants with the respective disease as numerator and the total number of participants as denominator.

To evaluate the quality of identified literature and the heterogeneity between different studies we used an evaluation score based on previously published studies [8]. For this score every included article was reviewed on sampling method, response rate, sample size, and data collection method. A maximum of five points would account for ‘high quality’, three to four points would be ‘moderate’ and zero to two would be ‘low quality’.

### Quantitative data synthesis

Study populations were divided into age ranges of childhood (0–10 years of age), adolescence (11–17 years), or adulthood (18–79 years). For each study, we extracted the prevalence rates of coexisting allergic rhinitis and asthma, as well as of allergic rhinitis only, and asthma only separately for male and female participants. We then calculated male–female ratios for each study, as well as pooled male–female-ratio estimates with 95% confidence intervals (95% CI) using random-effects meta-analyses with the inverse variance method (SR). Heterogeneity between the studies was measured by I^2^. Statistical analyses were done using Review Manager (RevMan), Version 5.3. Copenhagen (The Nordic Cochrane Centre, The Cochrane Collaboration, 2014) and R (R Foundation for Statistical Computing, Vienna, Austria).

## Results

### Characteristics of included studies

**Fig. 1:**
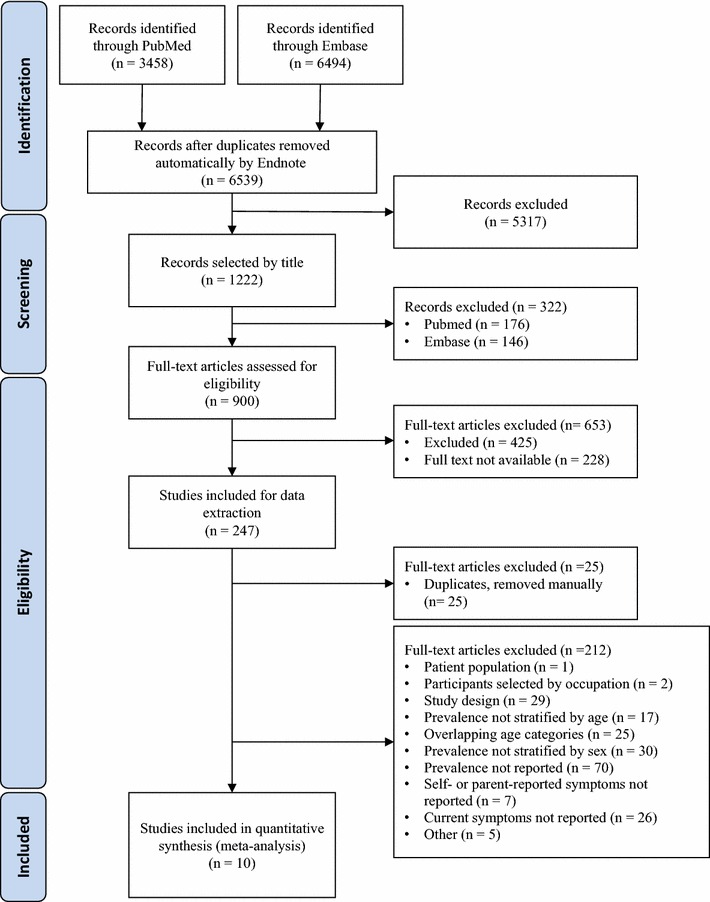
PRISMA flow chart for literature search

1222 out of 6539 publications were selected by title screening. Of those, 247 studies were eligible for data extraction since they reported prevalence of allergic rhinitis stratified by sex. Finally, 10 studies reporting the prevalence of asthma alone and allergic rhinitis with coexisting asthma were included into the systematic review (Fig. 1, Table 1) [8–16]. Six studies provided sex-specific prevalence of coexisting allergic rhinitis and asthma, allergic rhinitis only, and asthma only in children (0–10 years), two studies in adolescents (11–17 years), and two studies in adults (18–79 years). Studies with a broad age range were categorised as closely as possible to the targeted age groups, using the mean age of the participants for one study [16]. The assessment of allergic symptoms was questionnaire-based, mainly using the International Study of Asthma and Allergies in Childhood (ISAAC) [17] questionnaire for 8 studies in children and adolescents [8, 10–15, 18], or the European Community Respiratory Health Survey (ECRHS) [19] for 2 studies in adults [9, 20]. See Additional file 1: Tables E1–E3 for further description of study characteristics and Additional file 1: Table E5 for study results.

### Male–female ratios of coexisting allergic rhinitis and asthma

**Fig. 2:**
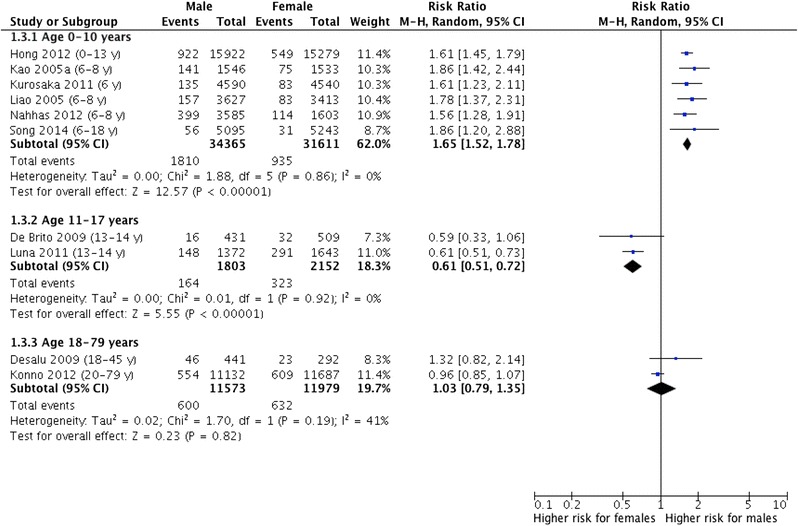
Forest plot estimating the difference in prevalence of current coexisting allergic rhinitis and asthma between males and females in childhood, adolescence and adulthood

We included 6 studies with a total of 34,365 males and 31,611 females for children (0–10 years), 2 studies with 1803 males and 2152 females for adolescents (11–17 years) and 2 studies with 11,573 males and 11,979 females for adults (18–79 years). The pooled estimates for the male–female ratio (males vs. females) of the prevalence of coexisting allergic rhinitis and asthma were 1.65 (95% CI 1.52–1.78) in children, 0.61 (0.51–0.72) in adolescents, and 1.03 (0.79–1.35) in adults (Fig. 2).

**Table 1:** Main characteristics of studies included in the systematic review

Study characteristics	Number of studies
Total	10
Study period	
2000–2007	7
2008–2014	3
Region	
Africa	1
Asia	7
South America	2
Sample size analysed	
< 1000	2
1001–5000	1
5001–10,000	3
10,001–100,000	4
Age category (in years)	
0–10	6
11–17	2
18–79	2
Urbanicity	
Urban	7
Rural/urban	1
Unclear/not reported	2
Method for assessing prevalence	
ISAAC questionnaire	8
ECHRS questionnaire	2

*ISAAC* International Study of Asthma and Allergies in Childhood, *ECRHS* European Community Respiratory Health Survey

The studies reported a male predominance of coexisting allergic rhinitis and asthma in children and a female predominance in adolescents. Desalu et al. [9] and Konno et al. [20] showed heterogeneous results for adulthood.

### Male–female ratios of allergic rhinitis without asthma

We included 5 studies with 29,775 males and 27,071 females for children (0–10 years), 2 studies with 1803 males and 2152 females for adolescents (11–17 years) and 2 studies that included 11,573 males and 11,979 females for adults (18–79 years). The pooled estimates for the male–female ratio of the prevalence of allergic rhinitis only were 1.25 (1.19–1.32) in children, 0.80 (0.71–0.89) in adolescents and 0.98 (0.74–1.30) in adults.

None of the studies reported a female predominance in the prevalence of allergic rhinitis only among children. In contrast both studies providing information on adolescents showed a female predominance. Concerning the prevalence among adults the two analysed studies again showed heterogeneous results.

### Male–female ratios of asthma without allergic rhinitis

**Fig. 3:**
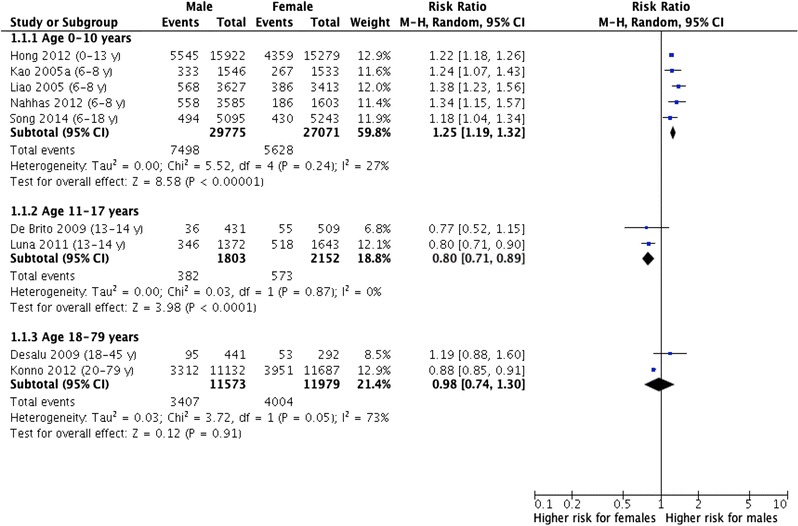
Forest plot estimating the difference in prevalence of allergic rhinitis only between males and females in childhood, adolescence and adulthood

We included 5 studies with 29,775 males and 27,071 females for children (0–10 years), 2 studies with 1803 males and 2152 females for adolescents (11–17 years). We found only one study providing information on asthma only in adults. The pooled estimates for the male–female-ratio of the prevalence of asthma only were 1.20 (0.99–1.45) in children and 1.03 (0.62–1.71) in adolescents. Konno et al. [20] reported in a study with 11,132 males and 11,687 females a male–female ratio for the prevalence of asthma only of 1.61 (1.44–1.81) in adults (Fig. 3).

Four of five included studies for asthma only in children reported a male predominance, whereas Nahhas et al. [15] showed a female predominance. Two studies analysed the prevalence of asthma only in adolescents and found heterogeneous results. De Brito et al. [8] reported a female predominance, whereas Luna et al. [14] showed a male predominance.

### Heterogeneity and quality of studies

While no statistical heterogeneity was detected among the lower age groups, moderate heterogeneity existed among the studies in the adult group (I_18–79_^2^ = 41%) for coexisting allergic rhinitis and asthma. In the meta-analysis for the prevalence of asthma only, considerable heterogeneity was found (I_0–10_^2^ = 75%; I_11–17_^2^ = 81%) resulting in an overall of I^2^ = 85%. Little or no heterogeneity was seen in studies reporting results for allergic rhinitis only in children and adolescents (I_0–10_^2^ = 27%; I_11–17_^2^ = 0%) compared to studies including adults (I_18–79_^2^ = 73%). All studies were of moderate quality (4 points) except from Desalu et al., which was rated as high quality (5 points), see Additional file 1: Table E4.

## Discussion

### Main findings

We found a clear ‘sex-switch’ in the prevalence of coexisting allergic rhinitis and asthma from a male predominance in childhood to a female predominance in adolescence. Similar trends of these sex-specific prevalence patterns were observed in participants with asthma only and those with allergic rhinitis only. Two studies in adults showed similar prevalence rates in both sexes.

### Comparison with other studies

In a global systematic review with meta-analysis we showed sex-related differences in rhinitis prevalence with a prevalence shift from a male predominance at around puberty to a female predominance thereafter [3]. Similarly, a retrospective analysis of the ECRHS data from 16 European countries showed a transition for asthma from a male predominance in childhood (0–10 years) followed by an equal gender distribution in adolescence (10–15 years) leading to a female predominance in adults (> 15 years) [21]. Sex-specific rhinitis and comorbid asthma prevalence data for older men and women are very scarce. Interestingly, according to a large observational all-female cohort, the Nurses’ Health Study in USA, the age-adjusted risk of asthma seems to be increased in postmenopausal women who ever or currently used hormone replacement therapy (i.e. conjugated estrogens with or without progesterone) compared to those who never used such hormones. However, allergic rhinitis with and without comorbid asthma has not been examinated [22]. In a cohort study of 509 children with allergic rhinitis from Turkey (mean age 7.2 ± 3.5 years, age range 1.5–18 years) Dogru showed that asthma was prevalent in the majority (53.2%) of these children [23]. In a French observational study of patients with asthma more than 50% of participants had concomitant allergic rhinitis [24]. Several narrative reviews showed this change in sex predominance favoring females during the transition from childhood to adulthood for diverse allergy-related diseases [4, 5, 25, 26]. Therefore, and since asthma and rhinitis coexist more often than expected [6], we hypothesized that also concomitant allergic rhinitis and asthma may undergo a similar sex-shift in prevalence during puberty.

Our results support this hypothesis to some extent. However, the limited number of studies found in adults did not allow us to clearly establish a clear tendency towards a male or female predominance but rather a balance between the sexes. Our pooled estimates relied only upon data from studies conducted in Asia (N = 7), South America (N = 2) and Africa (N = 1). In Pinart et al. a sex switch for allergic rhinitis prevalence around puberty was not found in studies conducted in Asia [3]. Five of six studies in the youngest age group (0–10 years) were from Asia, whereas no Asian studies were found for adolescents (11–17 years), suggesting a considerable bias.

Concerning possible mechanisms underlying a higher prevalence of allergic diseases in women during and after adolescence, higher levels of sex hormones such as estrogen and progesterone were suggested to be of central importance [27]. Sex hormones play a role in the homeostasis of immunity [28]. Estrogen and progesterone enhance type 2 and suppress type 1 responses in females, whereas testosterone suppresses type 2 responses in males [29]. Experiments in rodents showed an effect of estrogens on mast cell activation and the development of allergic sensitization, while progesterone can suppress histamine release but potentiate IgE induction [28]. Similarly for asthma sex differences have been reported for different phenotypes and symptom profiles in epidemiological, clinical and experimental studies, however, the aetiology remains largely unclear [30–33].

### Risk of bias

We tried to identify all population based studies reporting prevalence of coexisting allergic rhinitis and asthma. Given that such observational studies require large samples, it seems unlikely that a study of this dimension will have been published and not identified by our search. Furthermore, in population-based prevalence studies publication bias seems to be less of a concern than e.g. in interventional studies. Thus, we believe that a bias due to unpublished data is unlikely.

Our systematic review was embedded in a larger review considering the difference in prevalence for rhinitis only [3]. Although we used broad inclusion criteria, we may have missed studies that provided information on prevalence of having allergic rhinitis and asthma but did not provide information of having allergic rhinitis only or were published in journals that are not listed in the 2 major databases of medical literature, MEDLINE and EMBASE. Primary care-based studies including e.g. only out-patients were excluded because of a possible gender-related bias considering that women seek medical treatment, screening programs and other health care offers more often than men [34]. Restricting our search to studies published between 2000 and 2014 does not allow us to conclude on possibly different findings from earlier studies. The prevalence of allergies has dramatically increased in the second half of the twentieth century but reasons for these temporal trends are not clear [35]. We therefore wanted to avoid the rather speculative comparisons of prevalence studies across 5 and more decades and focused our evaluation on the 2 recent decades where the prevalence of allergies may have reached a plateau in many regions around the world [36].

Most of the included studies, especially in children, were conducted in Asia, which may limit the generalisability of the results, because of specific genetic differences between ethnic groups as well as different environmental factors for allergic diseases such as air pollution.

Our results showed that a sex switch from a male to a female predominance in the coexistence of allergic rhinitis and asthma is reported in population-based studies; however, further research is needed to study the underlying mechanisms. The definition of allergic rhinitis and asthma in our study is based on answers to validated questions from the ISAAC and ECRHS projects. Though these instruments are widely used globally and well validated in many languages especially for asthma, a possible overestimation of asthma or allergic rhinitis prevalence cannot be excluded. However, we do not think that this affects the male–female-ratios of the prevalence estimates used in our analysis since there is no indication for different overestimation between male and female responders of the included questions.

Using *current symptoms* of asthma and allergic rhinitis as outcome definition may cause misclassification if classifying individuals without symptoms because of successful symptom control for example as negative. However, we consider it unlikely that a person using e.g. anti-obstructive medication on a daily basis would answer negative to the question for having wheeze during the last 12 months. While, on the contrary, we judge the usage of doctor’s diagnosis to result in an underestimation of the number of subjects with allergic diseases.

Though there were many studies using the ISAAC study-design only few studies fulfilled our stringent inclusion criteria. This shows that there is a need for a more multimorbid perspective in population-based studies. For this work, we identified only cross-sectional studies. Although this study design is adequate for estimating population-based prevalences, longitudinal studies would be of interest to examine possible mechanisms underlying these differences in prevalence. Therefore, birth cohort studies in particular, are currently being evaluated regarding sex-specific allergy prevalence differences in childhood and early adolescence within the MeDALL project [37, 38].

Considerable inconsistency was found solely in our meta-analyses for asthma only and for the 2 adult studies as indicated by the Higgins’ I^2^-tests. These summary measures of the meta-analyses should be interpreted with extra caution. Potential sources of heterogeneity include study design, study area or analysed age groups, but the specific influence cannot be examined due to the limited number of studies.

## Conclusions

Based on a systematic review with meta-analysis of cross-sectional population-studies from across the globe we found a clear male predominance for the prevalence of coexisting allergic rhinitis and asthma in childhood. This seems to shift towards a female predominance in adolescents. Such a shift was less pronounced for allergic rhinitis as a single entity. Our results suggest that the effect of puberty seems to be particularly present in the most severely affected patients who have both allergic rhinitis and concurrent asthma. However, sex- and gender-specific evaluations beyond 14 years of age are scarce and further allergic multimorbidity studies in different population settings, particularly in adults, are required. In clinical, epidemiological and basic research more sex- and gender-specific analyses are needed to develop better prevention and treatment strategies.

## Additional file

**Additional file 1:** Search terms. Search terms and equations used for the original review [3] in PubMed and Embase databases. **Table E1** Characteristics of the included studies assessing prevalence of coexisting allergic rhinitis and asthma in children (0 – 10 y). **Table E2** Characteristics of the included studies assessing prevalence of coexisting allergic rhinitis and asthma in adolescents (11 – 17y). **Table E3** Characteristics of the included studies assessing prevalence of coexisting allergic rhinitis and asthma in adults (18 – 79 y). **Table E4** Study quality assessment of the included studies. **Table E5** Main results of included studies.

## Abbreviations

ECRHS: European Community Respiratory Health Survey
ISAAC: International Study of Asthma and Allergies in Childhood
PRISMA: Preferred Reporting Items for Systematic Reviews and Meta-Analyses
