# Supplementary material for: Is there a sex-shift in prevalence of allergic rhinitis and comorbid asthma from childhood to adulthood? A meta-analysis
Source: Clin Transl Allergy. 2017 Dec 5;7:44. doi: 10.1186/s13601-017-0176-5 (PMC5715620; doi:10.1186/s13601-017-0176-5)
Supplement: Supplementary file 1 — Additional file 1: Search terms. Search terms and equations used for the original review [3] in PubMed and Embase databases. Table E1 Characteristics of the included studies assessing prevalence of coexisting allergic rhinitis and asthma in children (0 – 10 y). Table E2 Characteristics of the included studies assessing prevalence of coexisting allergic rhinitis and asthma in adolescents (11 – 17y). Table E3 Characteristics of the included studies assessing prevalence of coexisting allergic rhinitis and asthma in adults (18 – 79 y). Table E4 Study quality assessment of the included studies. Table E5 Main results of included studies. [file 13601_2017_176_MOESM1_ESM.docx]

**Is there a sex-shift in prevalence of allergic rhinitis and comorbid asthma from childhood to adulthood?**

A metaanalysis.

*M Fröhlich, MD, MPH^1,2^, M Pinart, PhD^1,3,4,5,6^, T Keller, MSc^1^, A Reich^1^, B Cabieses, PhD^7^, C Hohmann, DiplPysch^1^, DS Postma, MD, PhD^8^, J Bousquet, MD, PhD^9,10^, JM Antó, MD, PhD^1,3,4,5^, T Keil, MD, MSc^1,11^, S Roll, PhD^1^*

1. Institute of Social Medicine, Epidemiology and Health Economics, Charité – Universitätsmedizin Berlin, Berlin, Germany
2. Clinic for Neonatology, Charité – Universitätsmedizin Berlin, Berlin, Germany
3. ISGlobal, Centre for Research in Environmental Epidemiology (CREAL), Barcelona, Spain
4. IMIM (Hospital del Mar Research Institute), Barcelona, Spain
5. Universitat Popmpeu Fabra (UPF), Barcelona, Spain
6. CIBER Epidemiología y Salud Pública (CIBERESP), Barcelona, Spain
7. Universidad del Desarrollo de Chile, Santiago, Chile
8. University of Groningen, University Medical Center Groningen, Department of Pulmonology, Groningen, The Netherlands
9. University Hospital, Montpellier, France
10. MACVIA-LR, Contre les Maladies Chroniques pour un Vieillissement Actifen Languedoc Roussillon, European Innovation Partnership on Active and Healthy Ageing Reference Site, and INSERM, VIMA: Ageing and Chronic Diseases, Epidemiological and Public Health Approaches, U1168, Paris, and UVSQ, UMR-S 1168, Université Versailles, St-Quentin-en-Yvelines, France
11. Institute of Clinical Epidemiology and Biometry, University of Wuerzburg, Wuerzburg, Germany

Layout

Search terms. Search terms and equations used for this review in PubMed and Embase databases 3

Table E1. Characteristics of the included studies assessing prevalence of coexisting allergic rhinitis and asthma in children (0 – 10 y) 4

Table E2. Characteristics of the included studies assessing prevalence of coexisting allergic rhinitis and asthma in adolescence (11 – 17y) 5

Table E3. Characteristics of the included studies assessing prevalence of coexisting allergic rhinitis and asthma in adults (18 – 79 y) 6

Table E4. Study quality assessment of the included studies 7

Table E5. Main results of included studies 8

References 9

# Search terms. Search terms and equations used for this review in PubMed and Embase databases

***Search strategy for Medline (Pubmed*) *in June 2014***

| Search | Query |
| --- | --- |
| #6 | Search (((((("Rhinitis, Allergic, Perennial "[Mesh] OR "Rhinitis, Allergic, Seasonal "[Mesh] OR Rhinitis[tiab] OR "Rhinitis"[Mesh:NoExp]))) AND ((Prevalence[tiab] OR incidence[tiab] OR “Prevalence”[MeSH] OR “Incidence”[MeSH] OR risk factors[tiab] OR "risk factors"[MeSH]))) AND ( "2000/01/01"[PDat] : "2014/12/31"[PDat] ))) NOT ((((("Rhinitis, Allergic, Perennial "[Mesh] OR "Rhinitis, Allergic, Seasonal "[Mesh] OR Rhinitis[tiab] OR "Rhinitis"[Mesh:NoExp]))) AND ((Prevalence[tiab] OR incidence[tiab] OR “Prevalence”[MeSH] OR “Incidence”[MeSH] OR risk factors[tiab] OR "risk factors"[MeSH]))) AND Review[ptyp] AND ( "2000/01/01"[PDat] : "2014/12/31"[PDat] )) |
| #5 | Search ((("Rhinitis, Allergic, Perennial "[Mesh] OR "Rhinitis, Allergic, Seasonal "[Mesh] OR Rhinitis[tiab] OR "Rhinitis"[Mesh:NoExp]))) AND ((Prevalence[tiab] OR incidence[tiab] OR “Prevalence”[MeSH] OR “Incidence”[MeSH] OR risk factors[tiab] OR "risk factors"[MeSH])) Filters: Review; Publication date from 2000/01/01 to 2014/12/31 |
| #4 | Search ((("Rhinitis, Allergic, Perennial "[Mesh] OR "Rhinitis, Allergic, Seasonal "[Mesh] OR Rhinitis[tiab] OR "Rhinitis"[Mesh:NoExp]))) AND ((Prevalence[tiab] OR incidence[tiab] OR “Prevalence”[MeSH] OR “Incidence”[MeSH] OR risk factors[tiab] OR "risk factors"[MeSH])) Filters: Publication date from 2000/01/01 to 2014/12/31 |
| #3 | Search ((("Rhinitis, Allergic, Perennial "[Mesh] OR "Rhinitis, Allergic, Seasonal "[Mesh] OR Rhinitis[tiab] OR "Rhinitis"[Mesh:NoExp]))) AND ((Prevalence[tiab] OR incidence[tiab] OR “Prevalence”[MeSH] OR “Incidence”[MeSH] OR risk factors[tiab] OR "risk factors"[MeSH])) |
| #2 | Search (Prevalence[tiab] OR incidence[tiab] OR “Prevalence”[MeSH] OR “Incidence”[MeSH] OR risk factors[tiab] OR "risk factors"[MeSH]) |
| #1 | Search ("Rhinitis, Allergic, Perennial "[Mesh] OR "Rhinitis, Allergic, Seasonal "[Mesh] OR Rhinitis[tiab] OR "Rhinitis"[Mesh:NoExp]) |

***Search strategy for Embase in June 2014***

| 1. Rhinitis.ti,ab. |
| --- |
| 2. exp perennial rhinitis/ |
| 3. exp seasonal rhinitis/ |
| 4. allergic rhinitis/ or Rhinitis.mp. or rhinitis/ or perennial rhinitis/ |
| 5. 1 or 2 or 3 or 4 |
| 6. (Prevalence or incidence).ti,ab. |
| 7. risk factors.ti,ab. |
| 8. (prevalence or incidence or risk factors).mp. |
| 9. 6 or 7 or 8 |
| 10. 5 and 9 |
| 11. limit 10 to yr="2000 - 2014" |

# Table E1. Characteristics of the included studies assessing prevalence of coexisting allergic rhinitis and asthma in children (0 – 10 y)

| **Study Reference**  **(N=10)** | **Country** | **Study characteristics** | **Residency** | **Method for assessing prevalence of allergic rhinitis** | **Sample analysed** | **Study period** | **Age** |
| --- | --- | --- | --- | --- | --- | --- | --- |
| Hong et al ^1^ | Korea | This study enrolled 38,201 children between 0 and 13 years of age from 34 elementary schools and 136 children’s daycare centers to participate in the ‘Seoul Atopy Friendly School’. | Urban | ISAAC Q | 31201 | 2010 | 0-9 |
| Kao et al ^2^ | Taiwan | The area studied was Taoyuan County, northern Taiwan. First-grade children (aged 6–8) or eighth-grade (aged 13–15) children were recruited. One-stage stratified cluster random sampling was used in this study. | U/NR | ISAAC Q | 6190 | 2002 | 6-8 |
| Kurosaka et al ^3^ | Japan | We studied the epidemiology of allergic symptoms in six-year-old children attending primary schools throughout Himeji City, Japan, during a two-year period from 2005 to 2006. Himeji City, situated close to the Seto Inland Sea, has a population of about 530,000. The southern area of the city is industrial, with steel manufacture predominating, the central area is urban with a large population, and the northern area is rural with farmlands. | Rural/  Urban | ISAAC Q | 11013 | 2006 | 6 |
| Liao et al ^4^ | Taiwan | A stratified random sampling method was used (47 schools were sampled randomly from the list of all elementary schools in Changhwa County by 16 districts, areas, and school size). In addition 4 areas in Changhwa county were classified for the comparison of the prevalence, including (1) the “TaiHao Petroleum Company area” with a lot of air-pollution due to a petrochemical company located in the northern part of the county; (2) the “XiZhou area” nearby a huge incinerator located in the southern part of the county; (3) the area of the coastal region; and (4) the rest of Changhwa County. | U/NR | ISAAC Q | 7040 | 2002 | 6-8 |
| Nahhas et al ^5^ | Saudi Arabia | A list of all government and private primary schools (aged 6-12y) in Madinah was obtained from the General Directorate of Education in the Madinah region. Schools were then stratified (according to the geographical area and sex) and a random sample of 38 schools (9 schools for girls and 29 for boys) was approached. | Urban | ISAAC Q | 5188 | 2008 | 6-8 |
| Song et al ^6^ | China | We obtained school lists from the Department of Education in Shijiazhuang city and prepared sampling frames of mixed sex schools with over 100 pupils in each school year. One school from each sampling frame was randomly selected to produce 10 schools for the study. Using the ISAAC written question- naire for asthma, rhinitis and eczema, we sent 12000 questionnaires to parents or guardians of 6–22-year-old schoolchildren during March to May in 2011. | Urban | ISAAC Q | 10338 | 2011 | 6-18; Mean: 10,2 |

ISAAC: International Study of Asthma and Allergies in Childhood; Q: questionnaire; U/NR: unclear/not reported.Table E2. Characteristics of the included studies assessing prevalence of coexisting allergic rhinitis and asthma in adolescence (11 – 17y)

| **Study Reference**  **(N=3)** | **Country** | **Study characteristics** | **Residency** | **Method for assessing prevalence of allergic rhinitis** | **Sample analysed** | **Study period** | **Age** |
| --- | --- | --- | --- | --- | --- | --- | --- |
| De Brito et al ^7^ | Brazil | A cross-sectional study, with two components: a study in prevalence and an inter-case study (rhinitis symptoms) with a comparison group (no rhinitis symptoms), based on information from questionnaires applied in phase 3 of ISAAC in Recife. | Urban | ISAAC Q | 940 | 2002 | 13-14 |
| Luna et al ^8^ | Brazil | In 2006, among the schools who had in their records a number ≤50 adolescents in the age range of the study, 29 were randomly selected, distributed among the six regional administrations, respecting the proportion of students aged 13-14y from every region and thus ensuring the sample is representative of a heterogeneous population. | Urban | ISAAC Q | 3015 | 2006-2007 | 13-14 |

ISAAC: International Study of Asthma and Allergies in Childhood; Q: questionnaire; U/NR: unclear/not reported.

# Table E3. Characteristics of the included studies assessing prevalence of coexisting allergic rhinitis and asthma in adults (18 – 79 y)

| **Study Reference**  **(N=2)** | **Country** | **Study characteristics** | **Residency** | **Method for assessing prevalence of allergic rhinitis** | **Sample analysed** | **Study period** | **Age** |
| --- | --- | --- | --- | --- | --- | --- | --- |
| Desalu et al ^9^ | Nigeria | The study area has 12 electoral wards, obtained from the state electoral commission. To select the participants, a multi-stage cluster sampling approach was used based on cluster equality and homogeneity. The electoral wards were defined as clusters and a sample frame containing the list of 12 clusters was constructed. Of these, 9 were selected by simple random sampling. A list of households was drawn up for each cluster and these were also randomly selected. | Urban | ECRHS Q | 733 | 2005-2006 | 18-45 |
| Konno et al ^10^ | Japan | A population-based, cross-sectional study was conducted with Japanese subjects 20–79 years of age, living in ten different areas of Japan. Detailed methods for selecting areas, participants were not described but a reference was provided. | Urban | ECRHS Q | 22819 | 2006-2007 | 20-79 |

ECRHS: European Community Respiratory Health Survey; Q: questionnaire.

# Table E4. Study quality assessment of the included studies

| **Study Reference** | **Representativity** | | | **Reliability** | **Quality rating score**  Max= 5 |
| --- | --- | --- | --- | --- | --- |
|  | **Sampling method**  Probability sampling (including: simple random, systematic, stratified g, cluster, two-stage and multi-stage sampling)=1  Non-probability sampling (including: purposive, quota, convenience and snowball sampling)=0 | **Sample size**  Calculated**=**1  Not calculated= 0 | **Response rate**  Reported=1  Not reported or if RR<60% =0 | **Data collection method**  ISAAC/ECHRS=2  Non-ISAAC/ECHRS but validated questionnaires=1  Non- validated questionnaires or Not clearly defined=0 |  |
| De Brito et al ^7^ | 1 | 1 | 0 | 2 | 4 |
| Desalu et al ^9^ | 1 | 1 | 1 | 2 | 5 |
| Hong et al ^1^ | 1 | 1 | 0 | 2 | 4 |
| Kao et al ^2^ | 1 | 0 | 1 | 2 | 4 |
| Konno et al ^10^ | 1 | 0 | 1 | 2 | 4 |
| Kurosaka et al ^3^ | 1 | 0 | 1 | 2 | 4 |
| Liao et al ^4^ | 1 | 0 | 1 | 2 | 4 |
| Luna et al ^8^ | 1 | 0 | 1 | 2 | 4 |
| Nahhas et al ^5^ | 1 | 0 | 1 (RR given for boys (86.2%) and girls (84.6%) | 2 | 4 |
| Song et al ^6^ | 1 | 0 | 1 | 2 | 4 |

# Table E5. Main results of included studies

| **Study** | **Rhinitis** | | | | | | | **Asthma** | | | | | | | **Rhinitis and Asthma** | | | | | | |
| --- | --- | --- | --- | --- | --- | --- | --- | --- | --- | --- | --- | --- | --- | --- | --- | --- | --- | --- | --- | --- | --- |
|  | male | | | female | | | MFR | male | | | female | | | MFR | male | | | female | | | MFR |
|  | n | N | % | n | N | % |  | n | N | % | n | N | % |  | n | N | % | n | N | % |  |
| De Brito et al ^7^ | 36 | 431 | 8.4 | 55 | 509 | 10.8 | 0.8 | 41 | 431 | 9.5 | 62 | 509 | 12.2 | 0.8 | 16 | 431 | 3.7 | 32 | 509 | 6.3 | 0.6 |
| Desalu et al ^9^ | 95 | 441 | 21.5 | 53 | 292 | 18.2 | 1.2 |  |  |  |  |  |  |  | 46 | 441 | 10.4 | 23 | 292 | 7.9 | 1.3 |
| Hong et al ^1^ | 5545 | 15922 | 34.8 | 4359 | 15279 | 28.5 | 1.2 | 503 | 15922 | 3.2 | 400 | 15279 | 2.6 | 1.2 | 922 | 15922 | 5.8 | 549 | 15279 | 3.6 | 1.6 |
| Kao et al ^2^ | 333 | 1546 | 21.5 | 267 | 1533 | 17.4 | 1.2 | 84 | 1546 | 5.4 | 62 | 1533 | 4.0 | 1.3 | 141 | 1546 | 9.1 | 75 | 1533 | 4.9 | 1.9 |
| Konno et al ^10^ | 3312 | 11132 | 29.8 | 3951 | 11687 | 33.8 | 0.9 | 682 | 11132 | 6.1 | 444 | 11687 | 3.8 | 1.6 | 554 | 11132 | 5.0 | 609 | 11687 | 5.2 | 1.0 |
| Kurosaka et al ^3^ |  |  |  |  |  |  |  |  |  |  |  |  |  |  | 135 | 4590 | 2.9 | 83 | 4540 | 1.8 | 1.6 |
| Liao et al ^4^ | 568 | 3627 | 15.7 | 386 | 3413 | 11.3 | 1.4 | 155 | 3627 | 4.3 | 109 | 3413 | 3.2 | 1.3 | 157 | 3627 | 4.3 | 83 | 3413 | 2.4 | 1.8 |
| Luna et al ^8^ | 346 | 1372 | 25.2 | 518 | 1643 | 31.5 | 0.8 | 127 | 1372 | 9.3 | 116 | 1643 | 7.1 | 1.3 | 148 | 1372 | 10.8 | 291 | 1643 | 17.7 | 0.6 |
| Nahhas et al ^5^ | 558 | 3585 | 15.6 | 186 | 1603 | 11.6 | 1.3 | 475 | 3585 | 13.2 | 237 | 1603 | 14.8 | 0.9 | 399 | 3585 | 11.1 | 114 | 1603 | 7.1 | 1.6 |
| Song et al ^6^ | 494 | 5095 | 9.7 | 430 | 5243 | 8.2 | 1.2 | 61 | 5095 | 1.2 | 42 | 5243 | 0.8 | 1.5 | 56 | 5095 | 1.1 | 31 | 5243 | 0.6 | 1.9 |

MFR = male-female-ratio

# References

1. Hong S, Son DK, Lim WR, et al. The prevalence of atopic dermatitis, asthma, and allergic rhinitis and the comorbidity of allergic diseases in children. *Environmental health and toxicology.* 2012;27:e2012006.

2. Kao CC, Huang JL, Ou LS, See LC. The prevalence, severity and seasonal variations of asthma, rhinitis and eczema in Taiwanese schoolchildren. *Pediatric allergy and immunology : official publication of the European Society of Pediatric Allergy and Immunology.* 2005;16(5):408-415.

3. Kurosaka F, Terada T, Tanaka A, et al. Risk factors for wheezing, eczema and rhinoconjunctivitis in the previous 12 months among six-year-old children in Himeji City, Japan: food allergy, older siblings, day-care attendance and parental allergy history. *Allergology international : official journal of the Japanese Society of Allergology.* 2011;60(3):317-330.

4. Liao MF, Huang JL, Chiang LC, Wang FY, Chen CY. Prevalence of asthma, rhinitis, and eczema from ISAAC survey of schoolchildren in Central Taiwan. *The Journal of asthma : official journal of the Association for the Care of Asthma.* 2005;42(10):833-837.

5. Nahhas M, Bhopal R, Anandan C, Elton R, Sheikh A. Prevalence of allergic disorders among primary school-aged children in Madinah, Saudi Arabia: two-stage cross-sectional survey. *PLoS One.* 2012;7(5):e36848.

6. Song N, Shamssain M, Zhang J, et al. Prevalence, severity and risk factors of asthma, rhinitis and eczema in a large group of Chinese schoolchildren. *The Journal of asthma : official journal of the Association for the Care of Asthma.* 2014;51(3):232-242.

7. Brito Rde C, da Silva GA, Motta ME, Brito MC. The association of rhinoconjunctivitis and asthma symptoms in adolescents. *Revista portuguesa de pneumologia.* 2009;15(4):613-628.

8. Luna Mde F, Almeida PC, Silva MG. [Asthma and rhinitis prevalence and co-morbidity in 13-14-year-old schoolchildren in the city of Fortaleza, Ceara State, Brazil]. *Cadernos de saude publica.* 2011;27(1):103-112.

9. Desalu OO, Salami AK, Iseh KR, Oluboyo PO. Prevalence of self reported allergic rhinitis and its relationship with asthma among adult Nigerians. *Journal of investigational allergology & clinical immunology.* 2009;19(6):474-480.

10. Konno S, Hizawa N, Fukutomi Y, et al. The prevalence of rhinitis and its association with smoking and obesity in a nationwide survey of Japanese adults. *Allergy.* 2012;67(5):653-660.
